# Systematic review searches must be systematic, comprehensive, and transparent: a critique of Perman et al

**DOI:** 10.1186/s12889-018-6275-y

**Published:** 2019-02-04

**Authors:** Devon Greyson, Ellen Rafferty, Linda Slater, Noni MacDonald, Julie A. Bettinger, Ève Dubé, Shannon E. MacDonald

**Affiliations:** 1 Vaccine Evaluation Center, British Columbia Children’s Hospital Research Institute & Department of Pediatrics, University of British Columbia, Vancouver, Canada; 2 5-308 Edmonton Clinic Health Academy, Faculty of Nursing, University of Alberta, Edmonton, AB T6G 1C9 Canada; 3 John W. Scott Health Science Library, University of Alberta, Edmonton, AB Canada; 4 Department Pediatrics, Dalhousie University, Halifax, NS Canada; 5 Institut national de santé publique du Québec, QC, Québec Canada

**Keywords:** Systematic review, Narrative review, Search methodology, School, Vaccination, Immunization, Scholarly communication

## Abstract

A high quality systematic review search has three core attributes; it is systematic, comprehensive, and transparent. The current over-emphasis on the primacy of systematic reviews over other forms of literature review in health research, however, runs the risk of encouraging publication of reviews whose searches do not meet these three criteria under the guise of being systematic reviews. This correspondence comes in response to Perman S, Turner S, Ramsay AIG, Baim-Lance A, Utley M, Fulop NJ. School-based vaccination programmes: a systematic review of the evidence on organization and delivery in high income countries. 2017; BMC Public Health 17:252, which we assert did not meet these three important quality criteria for systematic reviews, thereby leading to potentially unreliable conclusions. Our aims herein are to emphasize the importance of maintaining a high degree of rigour in the conduct and publication of systematic reviews that may be used by clinicians and policy-makers to guide or alter practice or policy, and to highlight and discuss key evidence omitted in the published review in order to contextualize the findings for readers. By consulting a research librarian, we identified limitations in the search terms, the number and type of databases, and the screening methods used by Perman et al. Using a revised Ovid MEDLINE search strategy, we identified an additional 1016 records in that source alone, and highlighted relevant literature on the organization and delivery of school-based immunization program that was omitted as a result. We argue that a number of the literature gaps noted by Perman et al. may well be addressed by existing literature found through a more systematic and comprehensive search and screening strategy. We commend both the journal and the authors, however, for their transparency in supplying information about the search strategy and providing open access to peer reviewer and editor’s comments, which enabled us to understand the reasons for the limitations of that review.

## Background

Our research team read with interest the article by Perman et al. [1] entitled *School-based vaccination programmes: a systematic review of the evidence on organization and delivery in high income countries.* The stated aim of the study was to identify and synthesize contextual and organisational factors that act as barriers or enablers to effective vaccine delivery in school-based settings. As we are involved in research regarding human papillomavirus (HPV) vaccination in Canadian schools, we were eager to discover a pre-existing synthesis of relevant literature on the topic; however, we were troubled to find that key literature on the topic was overlooked. As with other forms of empirical analysis, a systematic review can only be as strong as the foundational data collection on which it stands. As we examined the supporting documentation for the review, we became concerned that this case of a systematic review relying on incomplete data might not only mislead readers, but may be an example of a greater problem in scholarly publishing today.

The meteoric rise in the number of systematic reviews being conducted, and the inconsistent quality of these reviews, has been a topic of recent concern [2]. Concurrent with the increasing rate of published systematic reviews, other forms of literature review (e.g., narrative reviews) have fallen from favour, due to the assumption that systematic reviews are necessarily of higher (‘gold standard’) quality [3]. While systematic review search strategies and synthesis methods may vary depending on the subject matter and evidence type under examination, a high quality systematic review search has three core attributes; it is systematic, comprehensive, and transparent. Upon reviewing the Perman et al. [1] article and its accompanying supplemental and open peer review documents, we do not believe the article met these criteria. Specifically, we identified issues with the rigor of the search strategy and screening procedures. Furthermore, we noted limitations in the article review process that led to the misrepresentation of this article as a systematic review. Our objectives in this correspondence are threefold: (a) to note limitations in the systematic and comprehensive nature of the search strategy and article review process, and how this may have affected Perman et al.’s conclusions; (b) to note the way that the transparency of that published review and accompanying materials did provide critical information about the article review, revision, and acceptance process; and finally (c) to highlight and discuss key articles omitted in the original review, in order to contextualize the findings for readers.

## Lack of congruency with best practices in systematic reviews

Our primary concern is that the search strategy developed and implemented by Perman et al. [1], as reported in the supplemental materials, was neither systematic nor comprehensive enough for a systematic review. We find no indication that the authors employed the assistance of an information scientist or research librarian, whose specialized graduate training in knowledge organization, search and retrieval, and access to networks of expertise for search strategy peer review [4], would have strengthened the search, making it more systematic. In addition, the search keywords appeared potentially overly simplistic for a sophisticated data collection effort. Further, there was no rationale provided for restricting the search to just two databases, nor any description of additional data collection methods, such as systematic citation chaining or canvassing experts in the field, raising questions regarding how comprehensive this search could have been.

### Systematic and comprehensive search and screening

When we consulted our own research librarian, she helped us expand the published MEDLINE search to include all relevant terms for ‘school-based’ and ‘vaccination’, as well as the most relevant databases [5]. In the Table 1 below, we present the example MEDLINE search that the authors provided in their supplemental materials, along with a comparator search strategy developed by our research librarian, which resulted in an additional 1016 records. As seen in the Table 1, our proposed strategy did not include terms for children/adolescents, as this was deemed by our research librarian to excessively narrow the search. For instance, if the focus of the article was on nurse, staff or parent populations or policy issues, a relevant article may not include the term ‘child*’ or ‘adolescen*’. Steenbeek et al. [6] is one example of a highly relevant article that would have been excluded through use of the search terms child* and adolescen*.

**Table 1:** Number of articles identified when we applied the search strategy provided in Perman et al. [1] compared to that provided by our research librarian

Example of search strategy provided by Perman et al. [1]: conducted in Ovid MEDLINE^a^	Example of more comprehensive search strategy: conducted in Ovid MEDLINE
1. child* 2. adolescen* 3. school 4. exp. MASS VACCINATION 5. vaccination 6. 1 or 2 7. 4 or 5 8. 3 and 6 and 7 9. 8 with limits: Language (English), Publication Date (2000 to 2015) 10. Remove duplicates from 9	1. immunization/ or immunization schedule/ or vaccination/ or mass vaccination/ 2. (immuniz* or immunis* or vaccinat*).ti, ab, kf. 3. 1 or 2 4. schools/ or school health services/ or school nursing/ or “journal of school health”.jn. or (school* or kindergarten).ti, ab, kf. 5. 3 and 4 6. limit 5 to (english language and yr. = “2000–2015”) 7. Remove duplicates from 6
Identified 1659 records^b^	Identified 2675 records

^a^It was unspecified which database interface was used to search in the NICE portal, however, based on the search terms used (which are consistent with Ovid syntax), we undertook our comparison using Ovid MEDLINE

^b^We postulate the higher number of articles found in this search compared to Perman et al. [1] (*N* = 1139 in Figure 1) may be because their search was completed by August 2015, whereas ours included up to Dec 31 2015, and we could not fully replicate their search via the NICE portal

We also noted that Perman et al.’s [1] electronic search was limited to two databases (MEDLINE and Health Management Information Consortium [HMIC]). Mention is made of additional searches of the Cochrane Library, unspecified public health journals, reference lists of unspecified systematic reviews, and contacting one author. This would suffice for a narrative review, but is unlikely to be sufficiently comprehensive for a systematic review, particularly one focused on a question that is not purely biomedical or clinical in nature. The limited choice of databases searched (MEDLINE from the US and HMIC from the UK) likely resulted in failure to identify many European articles from locations other than the UK, as well as immunization program or policy literature published in other disciplines, which might have been captured in alternate databases [7]. This may have been exacerbated by the limited citation chaining and expert consultation, which could have helped point to relevant studies missed by the electronic search. Another potentially-useful addition to this particular review, given the topic, would have been a grey literature search, in order to capture program evaluation documents reported by health departments and school boards.

### Transparency

To ensure the transparency of systematic review research, it is important to provide sufficient documentation so that each step of the review process can be replicated. We commend Perman et al. [1] and the journal for their transparency in providing information about the search strategy, including their MEDLINE search. This information enabled our research librarian to partially replicate their search. However, there were other elements of the approach that were not explicated well enough for us to replicate. These include the use of undefined terms in the inclusion/exclusion criteria (e.g. “developed or high-income country”) and a lack of information on the reasons for article exclusion during screening. When we attempted to replicate this search and screening, we identified multiple articles published in or before August 2015 that were not included in the Perman et al.’s [1] review, but we believe met the inclusion criteria as outlined by the authors. These include articles by Gottvall et al. [8], Lind et al. [9], Ward et al. [10], Watson et al. [11] and Whelan et al. [12].

A strength of the review process was the journal’s open access to peer review and editor’s comments which enabled us to better understand the article review, revision, and acceptance process. We are of the opinion that Perman et al.’s [1] article is consistent with the authors’ original description of the article as a narrative review. The attempt to re-establish the article as a systematic review in order to fit the journal guidelines, however, resulted in misrepresentation of the article’s methodology. Furthermore, because the change in terminology appeared to occur after peer review, the methodology was never peer-reviewed for adherence to systematic review standards and procedures.

## Identified gaps in the literature may be addressed through an alternate search and screening strategy

Perman et al. [1] identified a number of alleged gaps in the literature based on their synthesis of studies included in their review. We argue that in some cases these gaps are partially addressed by existing literature identified though a more rigorous search strategy and screening procedure.

### Geographic representation

Perman et al. [1] note that the literature is “dominated by studies of pandemic and non-pandemic influenza vaccination in the US”. This is true of the literature they capture, with approximately 60% of the articles focusing on the US and 20% focusing on the UK. We found this somewhat surprising as there are many countries that have a more extensive history of offering vaccinations in schools than the US, including Canada and Australia. Without completing a full systematic review ourselves, we cannot determine if UK and US studies truly do dominate the literature. However, we did identify relevant literature that was not included in this systematic review from settings outside of the US and the UK. For example, Ward et al. [10] explored the history of adolescent vaccination in Australian schools, and identified key operational, implementation and procedural factors relevant to school-based immunization, as well as the impact of procedural changes on coverage across jurisdictions. Also from Australia, Braunack-Mayer et al. [13] identified ethical challenges for the delivery of adolescent immunization in a school-based setting, including informed consent and restrictions on privacy. Important Canadian literature in the field includes Steenbeek et al. [6] and Wilson et al.’s [14] findings on the HPV vaccine consent process in schools across Canada, as well as other Canadian literature [9, 12, 15].

Perman et al.’s [1] observation that European literature (other than the UK) was missed due to limiting their search to English language articles is also questionable. For instance, our English-only MEDLINE search identified three relevant articles from Sweden [8, 16, 17]. Inclusion of these additional Canadian, Australian and European articles would have allowed for comparison of the organizational factors that influence the implementation of school-based vaccination programs, including barriers and enablers, in different jurisdictions and geographical regions.

### The parental perspective

Perman et al. [1] identify a gap in the literature on the views of parents, children and adolescents on organizational factors of school vaccination programmes. However, our alternate search/screening strategy identified relevant articles from the parent perspective. These included Lind et al. [9], in which parents of children aged 5–8 were interviewed to explore their views on adding a school-based influenza immunization program and their opinions on the advantages, disadvantages and structuring of the program. Furthermore, Allison et al. [18] assessed parents’ perception of school-based influenza immunization. They identified benefits of schools versus medical settings such as fewer competing time demands, more convenience, and decreased cost, and found barriers to school settings, such as parents’ wish to be present for children’s vaccination and concerns about the competence of the person delivering the vaccines [18].

### Evaluation studies and measures of success

While a key literature gap described by Perman et al. [1] was school-based immunization program evaluations with specific measurements of success, we found examples of this type of evaluative research omitted from their review. Two Canadian studies evaluated the process of implementing a school-based immunization program [12, 14]. For instance, Wilson et al. [14] measured how different strategies in the implementation of a school-based program (e.g. having a public health nurse at the school, HPV education, and a thank-you note to the teachers) could impact HPV vaccine uptake. In the Australian context, Watson et al. [11] identified challenges they faced while implementing HPV vaccination in schools, as well as providing a detailed description of the impacts of the program on coverage rates. Other studies looked at the effectiveness of particular school-based organizational factors, such as the impact of immunization recalls on uptake [19].

## Limitations of our critique

It is important to note some of the limitations in our critique of the Perman et al. [1] study. First, we did not conduct a full systematic review and therefore can only present selected examples of the literature that were missed in the authors’ analysis. Second, we had limited information on their exclusion criteria and which articles were excluded; therefore, it was challenging to differentiate between articles that were missed through the search strategy and those excluded during screening. However, we would argue that based on Perman et al.’s [1] stated objectives, many of the articles presented in this critique should have been found during the search and included in the final analysis.

## Conclusions

While Perman et al. [1] ask a relevant research question and present some useful and interesting literature on the organization and delivery of school-based immunization programs, we remain concerned about the absence of systematic review best practices. We believe the lack of a comprehensive and systematic search and screening process has omitted relevant articles, and therefore led to potentially incorrect conclusions being drawn based on incomplete data. We are of the opinion that the methods employed in this article are more consistent with its original presentation as a narrative review and the article should not have been reconstructed as a systematic review late in the process. However, we applaud both the journal and the authors for their transparency in publishing the supplemental and editorial/peer review documents, which allowed us to understand the search strategy and the article review and revision process. We hope that by noting some of the characteristics and limitations of this systematic review and the review process, we will draw attention to the need for rigour and demonstrate some of the pitfalls in not complying with best practice guidelines when conducting and publishing systematic reviews. We also hope to inform users and readers of the Perman et al. [1] article of additional research available on the organization and delivery of school-based immunization programs, in order to guide future work in this area.

## Response to: “Systematic review searches must be systematic, comprehensive, and transparent: A critique of Perman et al”

Sarah Perman^1^ sarah.perman@phe.gov.uk, Simon Turner^2^ s.turner@manchester.ac.uk, Angus I. G. Ramsay^3^ angus.ramsay@ucl.ac.uk, Abigail Baim-Lance^4^ abigail.baimlance@gmail.com, Martin Utley^5^ m.utley@ucl.ac.uk, Naomi J. Fulop^3^ n.fulop@ucl.ac.uk

^1^Public Health EnglandWellington House, 133–155 Waterloo Road, London, SE1 8UG, UK.

^2^Health Organisation, Policy and Economics (HOPE) research group, Centre for Primary Care, Division of Population Health, Health Services Research and Primary CareUniversity of ManchesterWilliamson Building, Oxford Road, Manchester, M13 9PL, UK.

^3^Department of Applied Health ResearchUniversity College London1–19 Torrington Place, London, WC1E 7HB, UK.

^4^Graduate School of Public Health and Health PolicyCity University New York55 W 125th St, New York, NY 10027, USA.

^5^Clinical Operational Research UnitUniversity College London4 Taviton Street, London, WC1H 0BT, UK.

We have been invited by the editors of BMC Public Health to respond to correspondence in relation to our research article [1]. We thank Greyson et al. for their response to our article suggesting that our study did not meet three quality criteria for systematic reviews, namely being “systematic, comprehensive and transparent”, and thereby calls into question the reliability of its findings. We make the following points in response to the correspondence:

## Suggestions for improving review methods

Greyson et al. make a number of useful suggestions for improving the conduct of systematic reviews, e.g. employing an information scientist /research librarian, using systematic citation chaining, and canvassing experts in the field. They state that by employing different search terms and conducting searches on additional databases we would have generated additional studies for our review. We accept that our search terms and strategy could have been improved and we will certainly reflect on the advice they give combined with other recent research on literature searching. That said, it is broadly accepted that systematic reviews can rarely be (or be proved to be) exhaustive, and so assessments of comprehensiveness come down to the added value of expanded searches.

Greyson et al. suggest that we inappropriately excluded studies identified through our search. In response to the authors’ correspondence we have re-run our searches on Medline via the National Institute for Clinical Excellence (NICE) Healthcare Databases Advanced Search (HDAS) portal in order to validate our results (https://hdas.nice.org.uk/.). We found that we had wrongly excluded one study because the researcher mistakenly concluded that the publication – a government communicable disease report - was not peer reviewed [10]. However, six of the seven studies that Greyson report finding when they replicated our search on Ovid Medline are not catalogued on the HDAS version of Medline which is provided by ProQuest. This suggests there may be some value in searching the same databases through different available portals.

## Selection of criteria for evaluating systematic reviews

Greyson et al. use our article to draw broader conclusions on the conduct of systematic reviews. No evidence is presented to support Greyson et al.’s choice of criteria for evaluating systematic reviews, suggesting these are based on the authors’ beliefs or experiences. In suggesting these criteria, there is no reference to published guidance on conducting systematic reviews, for example, the *Cochrane Handbook for Systematic Reviews of Interventions* [20]. We are not sure how useful it is to evaluate our form of review (and draw wider lessons on the conduct of systematic reviews) by choosing and applying criteria that are not related explicitly to established guidance on the standards for conducting such reviews.

We encourage the authors to develop these suggestions (and others) further and present them in a systematic format, e.g. as a set of questions to consider when planning review searches, and relate these to different types of review as appropriate.

In systematic reviews a wide array of methods are deployed including quality assessment, data extraction and data synthesis. While we accept the suggestions for improvement in our search methods, we maintain that the methods we used in other areas were reliable and consistent with accepted guidance for conduct of reviews [21]. Our methods were subject to quality assurance internally within our group and externally by peer reviewers, and are described in the methods section of our published paper.

## Forms of systematic review

Greyson et al. imply that only one form of systematic review exists and do not allow for the variety of forms of review that fall under this umbrella term. Specifically, we opted for a narrative synthesis approach. Narrative synthesis is a form of analysis accepted as having value within systematic reviews that span diverse forms of evidence. Scoping reviews are another accepted form of systematic review with their focus on identification of the main concepts and theories on a given topic. In our paper we cited the relevant guidance [21] for our narrative synthesis approach.

## Applying PRISMA guidance

In the decision letter following peer review, we were asked to address the following: “We note that you have carried out a narrative synthesis of the literature. As we only consider either systematic reviews, meta-analyses or scoping reviews, we ask that you please re-write your manuscript as a systematic review adhering to the PRISMA guidelines.” In response to this editorial comment, we applied the PRISMA guidelines to our review and were able to confirm that our review methods met the standards required in PRISMA guidance (http://www.prisma-statement.org/). The editorial process helped to shape how our review was presented. At a system level, potential preferences for systematic reviews over other forms of review, and the consequences of this for framing both research object and methods, could be explored further.

## Our review findings

We accept that a different search strategy may have generated different results. However, aside from some articles on parents’ perspectives on school-based vaccination, Greyson et al. have not presented new findings from the additional studies that they identified. We maintain that there are significant gaps in the research literature. These include an absence of theory-informed studies, a lack of high quality evaluations, and few studies which explore the views of children and teenagers. Our narrative synthesis of the studies that we identified in our systematic review generated important material on common organisational factors that influence the implementation of school based vaccination programmes. Greyson et al. have not presented material that alters these findings.

## Abbreviation

HPV: Human papillomavirus
